# Large Spin Hall Efficiency and Current‐Induced Magnetization Switching in Ferromagnetic Heusler Alloy Co_2_MnAl‐Based Magnetic Trilayers

**DOI:** 10.1002/advs.202407171

**Published:** 2024-12-04

**Authors:** Mingzhi Wang, Chang Pan, Nian Xie, Xuepeng Qiu, Yufei Li, Lili Lang, Shiqiang Wang, Dashuai Cheng, Weijia Fan, Shi‐Ming Zhou, Zhong Shi

**Affiliations:** ^1^ Shanghai Key Laboratory of Special Artificial Microstructure Materials and School of Physics Science and Engineering Tongji University Shanghai 200092 China; ^2^ State Key Laboratory of Materials for Integrated Circuits Shanghai Institute of Microsystem and Information Technology Chinese Academy of Sciences Shanghai 200050 China; ^3^ Key Laboratory of Materials Physics Institute of Solid State Physics Chinese Academy of Sciences Hefei 230031 China

**Keywords:** heusler alloy, spin–orbit torque, spintronics

## Abstract

The spin Hall efficiency (*ξ*) is a crucial parameter that evaluates the charge‐to‐spin conversion capability of a material, and thus materials with higher *ξ* are highly desirable in spin–orbit torque (SOT) devices. Recent studies have highlighted the use of ferromagnetic materials as robust spin sources, paving the way for the development of more efficient SOT devices. To accelerate this innovation, it is essential to pursue ferromagnetic materials of high *ξ*. Here the experimental observation of a large spin Hall efficiency is reported in ferromagnetic Heusler alloy Co_2_MnAl (CMA)‐based magnetic trilayers. Utilizing the current‐induced hysteresis loop shift technique, the spin Hall efficiency is determined to be 0.077 for the *B*2‐phase and 0.029 for the disordered CMA. Notably, magnetization switching both with and without the application of an external auxiliary magnetic field were achieved in these trilayers. The enhancement of *ξ* is attributed to the formation of chemical ordering in CMA. These findings provide new avenues for the development of ferromagnet‐based SOT devices.

## Graphical abstract

Large spin Hall efficiency (*ξ* = 0.077) and magnetization switching, which are favorable for effective spin–orbit torque applications, are achieved in Heusler alloy Co_2_MnAl‐based trilayers. These effects result from the strong spin Hall effect and can be further enhanced through chemical ordering, providing a promising direction for the development of spin–orbit torque devices utilizing ferromagnetic ordered alloys as a reliable spin source.

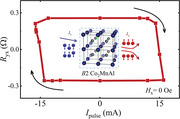

## 1 Introduction

Heusler alloys are a large family of ternary intermetallic compounds that have drawn considerable attention since their first discovery by Fritz Heusler over a century ago. Up to now, efforts have been made to investigate this remarkable material family and found extraordinary intrinsic properties such as topological superconductivity,^[^ ^1^, ^2^ ^]^ topological Dirac and Weyl band structure,^[^ ^3^, ^4^, ^5^, ^6^ ^]^ half‐metallicity,^[^ ^7^, ^8^, ^9^ ^]^ and beyond. Due to their high Curie temperature,^[^ ^9^, ^10^ ^]^ high spin polarization, and low magnetic damping constant,^[^ ^11^, ^12^, ^13^ ^]^ Co‐based full‐Heusler alloys are considered potential candidates for high‐performance spintronic applications. Moreover, intriguing transport effects are expected in Co‐based Heusler alloys. For example, the giant anomalous Hall effect (AHE) has been shown to be dominated by the intrinsic contribution arising from the topological Weyl semi‐metallicity,^[^ ^14^ ^]^ despite the influence of magnetization.^[^ ^15^, ^16^, ^17^ ^]^ As a typical representative of Co‐based full‐Heusler alloys, Co_2_MnAl (CMA) is currently drawing significant attentions as the topological Weyl semimetal band structure was predicted.^[^ ^18^ ^]^ The intrinsic contribution of AHE can be expressed in terms of the Berry curvature:

(1) $$\sigma_{\mathrm{xy}}=-\frac{e^{2}}{\hbar }\sum_{n}\int_{\mathrm{BZ}}\frac{dk}{{\left(2\pi \right)}^{3}}f_{kn}\Omega_{n}\left(k\right)$$

where *σ*_xy_ is the anomalous Hall conductivity (AHC), Ω_n_(**k**) is the Berry curvature, *f*_**k**n_ is the Fermi distribution function, *e* is the electron charge and ℏ is the reduced Planck constant. Numerous studies have suggested that the topology of the electronic band structure can result in a strong enhanced Berry curvature, which can significantly strengthen the AHC. In this regard, the topological band structure of CMA may also enhance the anomalous Nernst effect and spin Hall effect (SHE) through intrinsic contribution.^[^ ^19^ ^]^ For the former, the Mott relation describes the intrinsic correlation between the anomalous Nernst conductivity (ANC) and the derivative of AHC, and has been demonstrated in various materials. As for the latter, the SHE shares the same physical origin as the AHE, except for the fact that the charge current in heavy metals is non‐polarized, while it is polarized in ferromagnet. Thus, spin‐dependent scattering generates a pure spin current in heavy metals, whereas a voltage is detectable in ferromagnet. Recently, large AHC and ANC have been experimentally determined in CMA, in good agreement with theoretical calculations, which suggesting that the electric and thermoelectric conductivity are mainly due to the intrinsic contribution.^[^ ^20^, ^21^ ^]^ Therefore, large intrinsic spin Hall conductivity (SHC) is also expected in CMA, as theoretical calculations have predicted.^[^ ^16^, ^22^ ^]^ However, the experimental evidence of large SHC in CMA is still lacking.

Over the past decade, spin–orbit torque (SOT) has emerged as a promising candidate for nonvolatile memories and logic devices.^[^ ^23^ ^]^ To achieve more effective manipulation of magnetization and lower power consumption, materials with higher spin Hall efficiency are desired. Initially, studies mainly focused on heavy metals (HMs) due to their natural large spin–orbit coupling. However, recent researches have found that ferromagnetic materials also exhibit substantial spin Hall efficiency,^[^ ^24^, ^25^, ^26^, ^27^, ^28^, ^29^, ^30^, ^31^ ^]^ offering exciting prospects for mechanism study and application. Previous investigations on ferromagnets (FMs) primarily focused on the SOT generated by polycrystalline or those composed of heavy metals.^[^ ^26^, ^27^, ^28^, ^29^, ^30^, ^31^, ^32^, ^33^, ^34^, ^35^, ^36^, ^37^, ^38^, ^39^ ^]^ The generation of spin current has been attributed to the interfacial effects^[^ ^38^, ^40^, ^41^ ^]^ such as spin–orbit precession, spin–orbit filtering, and bulk effects such as SHE,^[^ ^26^, ^29^, ^30^ ^]^ AHE,^[^ ^31^, ^36^, ^37^, ^42^, ^43^ ^]^ and structural gradient.^[^ ^39^ ^]^ Recently, FMs have demonstrated promise as robust spin sources in magnetic FM/Ti/FM trilayer systems.^[^ ^35^, ^38^ ^]^ Thanks to the potential large SHC predicted in the CMA, significant interest has been sparked to obtain a large spin Hall efficiency (*ξ*) this Heusler alloy, which requires further experimental investigation urgently. In this study, we leverage the Co‐base Heusler alloy trilayers and observe a large spin Hall efficiency in Heusler alloy CMA. Moreover, by comparing the *B*2 ordered and disordered CMA, we address that the ordered CMA is of higher potential in spin generation and that the chemical ordering is of great importance in ferromagnetic Heusler alloys on SOT application. Notably, the absence of heavy metals in CMA is of particular significance, as it facilitates a thorough understanding of the effects brought about by chemical ordering.

We conduct a systematic investigation of SOT in ferromagnetic Heusler alloy CMA‐based magnetic trilayers, focusing on the CMA/Ti/Co_40_Fe_40_B_20_ (CFB)/MgO/SiO_2_ multilayer. Through the current‐induced hysteresis loop shift technique,^[^ ^44^ ^]^ we have determined a higher efficiency (*ξ* = 0.077) in *B*2‐ordering CMA (referred to as *B*2 CMA) on SOT generation compared to the disorder phase CMA (*ξ* = 0.029). The current‐induced magnetization switching was further proved, which the spin–orbit torque is generated from CMA. Our findings not only underscore the robustness of SOT in the magnetic trilayers but also highlight the promising prospects for Heusler alloys in broader applications.

## 2 Results and Discussion

### 2.1 Material Structure Characterization

**Figure 1:**
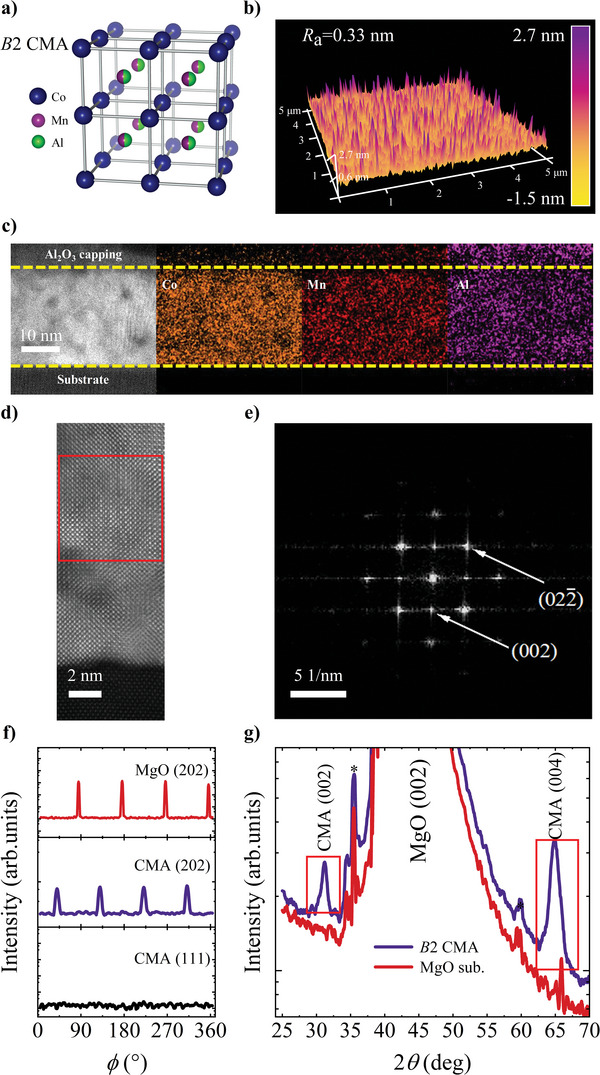
Structure characterization of *B*2 CMA. a) Schematic representation of the crystal structure of *B*2 CMA. b) AFM image of the 20 nm *B*2 CMA single layer. c) EDS mapping of Co, Mn, and Al elements in *B*2 CMA film. Between the yellow dashed lines is the region of CMA. d) Cross‐sectional STEM image of CMA taken along the [100] zone axis. e) FFT of the region in red square of d). f) Pole figures of MgO (202), CMA (202), and CMA (111) with Chi  =  45 ^○^ and 54.74 ^○^, respectively. The absence of CMA (111) indicates the CMA is *B*2 ordered. g) XRD Bragg diffraction patterns of the reference MgO (001) substrate and *B*2 CMA. The minor peaks marked by asterisk are coming from MgO substrates.

**Figure** **1**a shows the crystal structure of *B*2‐ordered CMA, where Mn and Al atoms are indistinguishable. The AFM image of the *B*2 CMA film is displayed in Figure 1b. The analysis reveals that the single‐layer CMA possesses an average roughness *R*_a_ = 0.33 nm, indicating a smooth film surface. The energy dispersive spectrometer (EDS) mapping of *B*2 CMA single layer with Al_2_O_3_ capping shown in Figure 1c displays uniform distribution of Co, Mn, and Al elements. The composition is further determined to be Co: Mn: Al = 54: 23: 23. Figure 1d shows the scanning transmission electron microscopy (STEM) image. The clear periodic distribution of atoms and the corresponding selected‐area diffraction pattern verify an excellent formed *B*2‐structure. In addition, compared with MgO substrate, two additional diffraction peaks appear at 2*θ*  =  31.23 ^○^ and 64.91 ^○^ in the X‐ray diffraction (XRD) patterns of *B*2 CMA as presented in Figure 1f, representing the CMA (002) and CMA (004) lattice planes, respectively. The pole figures (*ϕ*scan) displays two sets of peaks representing the CMA (202) and MgO (202) lattice plane at Chi  =  45 ^○^. Notably, the absence of CMA (111) peaks at Chi  =  54.74 ^○^ along with the appearance of fourfold CMA (202) peaks suggests that a *B*2‐structure has formed in the CMA film, where random distribution occurs between Mn and Al atoms, rather than the fully ordered *L*2_1_‐phase. Furthermore, the set of CMA (202) peaks deviates from the MgO (202) peaks by *ϕ*  =  45°. This observation implies a particular epitaxial growth relationship, wherein the [100] crystal axis of MgO aligns parallel to the [110] axis of CMA.

### 2.2 Current‐Induced Magnetization Switching Under Auxiliary Magnetic Field

**Figure 2:**
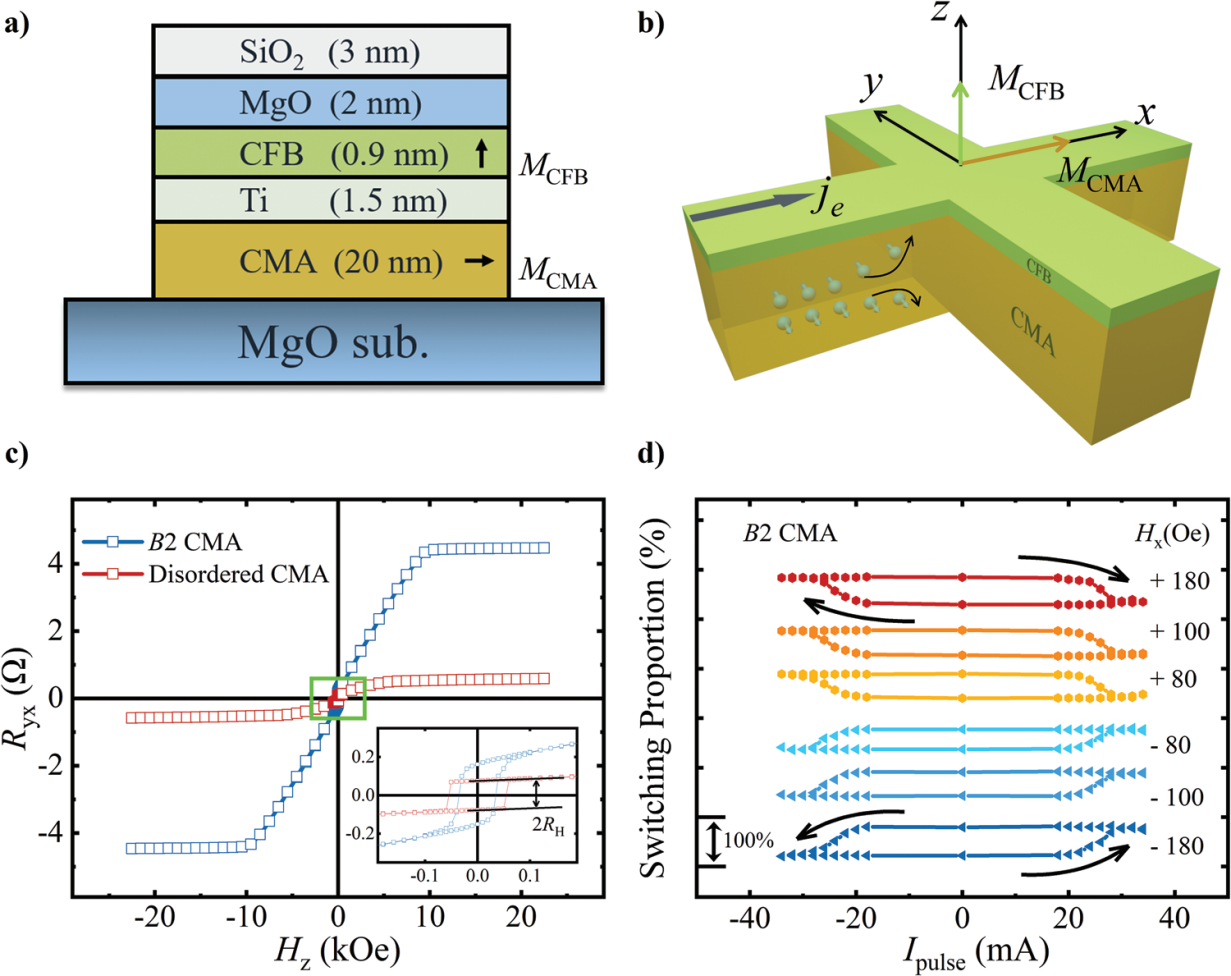
a) Illustration of MgO(substrate)/CMA (20 nm)/Ti (1.5 nm)/CFB (0.9 nm)/MgO (2 nm)/SiO_2_ (3 nm) multilayer, where the black arrows indicate the direction of magnetization. b) Schematic illustration of the Hall bar for measurement. c) The AHE loops of multilayers with *B*2 and disordered CMA, respectively. The inset is the enlarged area of the green rectangle box. d) Current‐induced magnetization switching under different auxiliary magnetic field ranging from + 180 Oe to − 180 Oe.

**Figure** **2**a illustrates the structure of the multilayer used for the measurement, which consists of CMA (20 nm)/Ti (1.5 nm)/CFB (0.9 nm)/MgO (2 nm)/SiO_2_ (3 nm). The black arrows indicate the magnetization direction of CFB and CMA, with CFB being perpendicular and CMA being parallel to the film plane, respectively. Figure 2b depicts the sketch of the Hall bar we used in the measurement, where the charge current flows in the x‐direction, and the spin current generated by CMA flows in the z‐direction. In Figure 2c, the inset shows the low‐field square loops of *R*_yx_, which represent the anomalous Hall resistance from CFB, as a result of the establishment of perpendicular magnetic anisotropy (PMA) in CFB. As the field increases, a significant linear increase in *R*_yx_ is observed, saturating at H = ± 8 and 10 kOe, which can be attributed to the coherent rotation of magnetization in the disordered and *B*2 CMA toward the out‐of‐plane hard axis, respectively. The intermediate Ti layer was introduced to decouple the magnetization of CFB and CMA layers and develop the PMA of CFB. Remarkably, it is unambiguously that anomalous Hall resistance of *B*2 CMA is larger than the disordered counterpart in Figure 2b. To gain a deeper understanding of the AHE in CMA, we calculate the anomalous Hall conductivity using the formula $\sigma_{\mathrm{xy}}=\rho_{\mathrm{yx}}/\rho_{\mathrm{xx}}^{2}$, where $\rho_{\mathrm{xx}}^{B2\;\mathrm{CMA}}=271.26\;\mu \Omega \cdot \mathrm{cm}$, $\rho_{\mathrm{yx}}^{B2\;\mathrm{CMA}}=9.33\;\mu \Omega \cdot \mathrm{cm}$, $\rho_{\mathrm{xx}}^{\mathrm{Disordered}\;\mathrm{CMA}}=146.75\;\mu \Omega \cdot \mathrm{cm}$, and $\rho_{\mathrm{yx}}^{\mathrm{Disordered}\;\mathrm{CMA}}=0.90\;\mu \Omega \cdot \mathrm{cm}$. These values are determined by excluding the shunting effect. As a result, the value of $\sigma_{\mathrm{xy}}^{B2\;\mathrm{CMA}}=126.80\;S\cdot {\mathrm{cm}}^{-1}$ is not relevant to CFB and is over three times the value observed in the disordered counterpart $\sigma_{\mathrm{xy}}^{\mathrm{Disordered}\;\mathrm{CMA}}=41.79\;S\cdot {\mathrm{cm}}^{-1}$. Hence, we emphasize the significantly elevated AHC in *B*2 CMA, attributing it to the influence of chemical ordering on the electronic and magnetic properties of the Heusler alloy.

To study the magnetization switching behavior, we conducted measurements under different auxiliary magnetic field, as shown in Figure 2d. The auxiliary magnetic field is set in the same direction as the pulse current.^[^ ^38^ ^]^ The switching proportion parameter is defined by $\delta =\frac{R_{\mathrm{switching}}}{2R_{H}}\times 100\%$, where *R*_switching_ is the transverse resistance during switching process, and *R*_H_ is the value of anomalous Hall resistance of CFB as shown in Figure 2c. When comparing the case where a positive auxiliary magnetic field is applied, it is evident that the switching behavior completely reversed when a negative auxiliary magnetic field is employed (The same outcome holds true for disordered CMA, see supporting information). Considering the positive AHE observed in CFB as depicted in Figure 2c, it indicates that the effective spin Hall efficiency of CMA is positive, consistent in sign with that of ferromagnet NiFe and opposite to the non‐magnetic metal Ta, ferromagnet CFB^[^ ^38^ ^]^ and Co_2_MnGa (CMG).^[^ ^45^, ^46^ ^]^

### 2.3 Magnetization‐Dependent Field‐Free Switching and Evaluation of Spin Hall Efficiency

**Figure 3:**
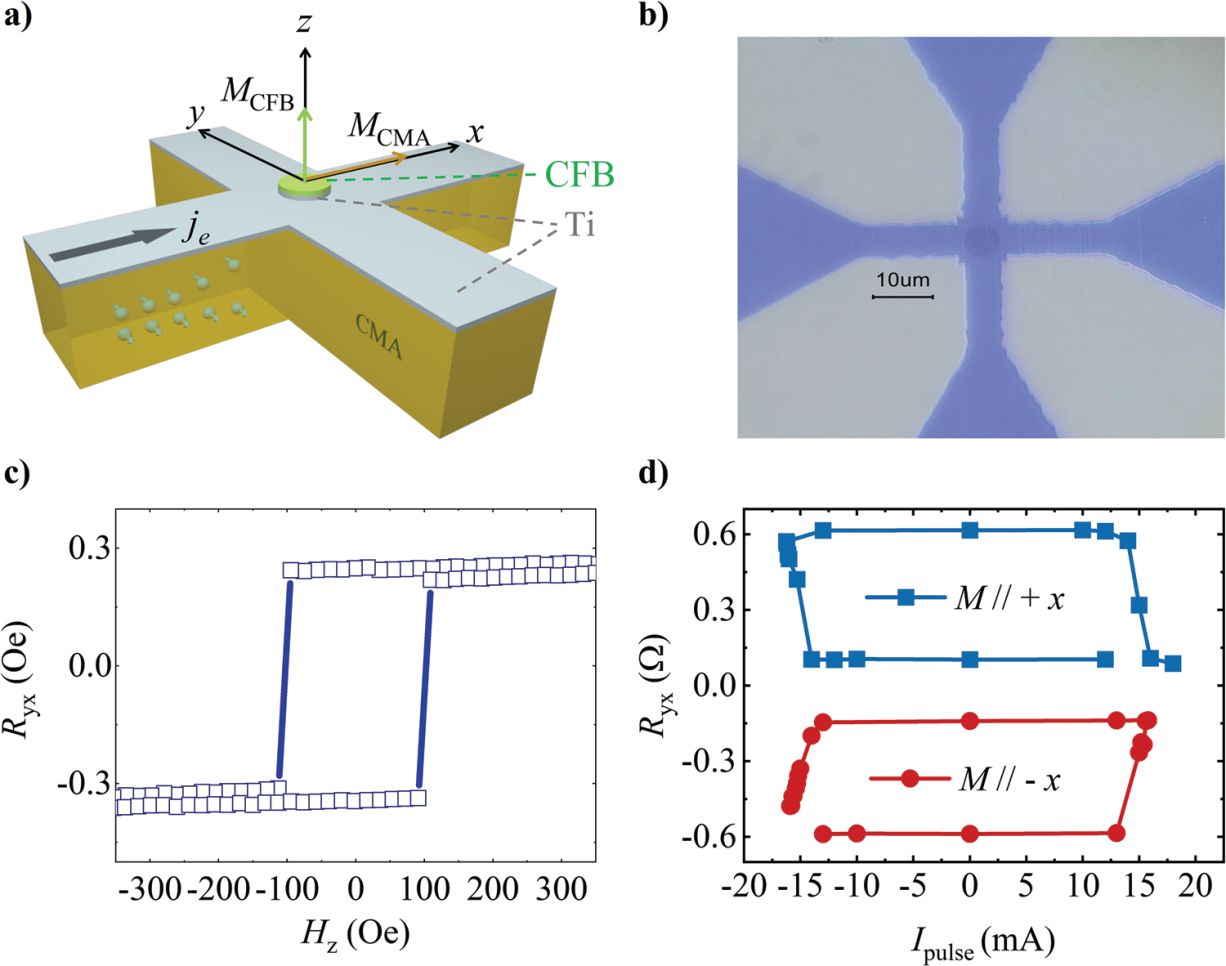
a) Schematic illustration of the pillar structure. b) Optical microscope image of the device. The central pillar reserves the complete CMA/Ti/CFB/MgO/SiO_2_ multilayer. The remainder of the Hall bar consists of only CMA/Ti layers. c) Anomalous Hall resistance results of the device, showing a square loop that indicates good PMA in the central pillar. d) Current‐induced magnetization switching without an auxiliary magnetic field. The polarity of the magnetization switching is reversed when the M_CMA_ is aligned to the + x or − x direction. The switching loops are vertically shifted for clarity.

We further observed a notable proportion of field‐free switching in both the *B*2 and disordered CMA trilayers. The polarity of switching can be successfully reversed by aligning the magnetization of CMA in the ± x direction without any auxiliary field, indicating a magnetization‐dependent *z* **‐**spin. Meanwhile, the field‐free switching proportion is not as large as the switching proportion with an auxiliary field (see Figure S1.2, Supporting Information), and the loop shift at *H*_x_ =  0 Oe is almost absent. We note that the small switching proportion may arise from the square shape of our Hall bar, which causes the redistribution of electric current.^[^ ^47^ ^]^ Additionally, the sample exhibits non‐uniform perpendicular magnetization, with certain regions being more difficult to switch via spin–orbit torque than others.^[^ ^48^ ^]^ Nonetheless, recent progress demonstrates that optimizing the shape, specifically using a round pillar, can substantially increase the switching proportion.^[^ ^49^, ^50^ ^]^ Hence, to reduce the edge effect and provide a more comprehensive demonstration of the *z*‐spin behavior in *B*2 CMA, we further optimized the configuration and constructed a pillar device. The pillar is etched down to the Ti layer, preserving the entire *B*2 CMA/Ti/CFB/MgO/SiO_2_ multilayer, while only *B*2 CMA and partial Ti remain in the rest of the Hall bar as illustrated in **Figure** **3**a. The radius of the pillar in our *B*2 CMA (10)/Ti (1.5)/CFB (0.9) trilayer is ≈3 µm, as shown in the optical microscope image in Figure 3b. Through device engineering, the measurement accuracy was significantly enhanced, achieving a field‐free switching ratio of up to 90%, as shown in Figure 3d. Meanwhile, the sample retained good PMA, as confirmed by the AHE results in Figure 3c.

**Figure 4:**
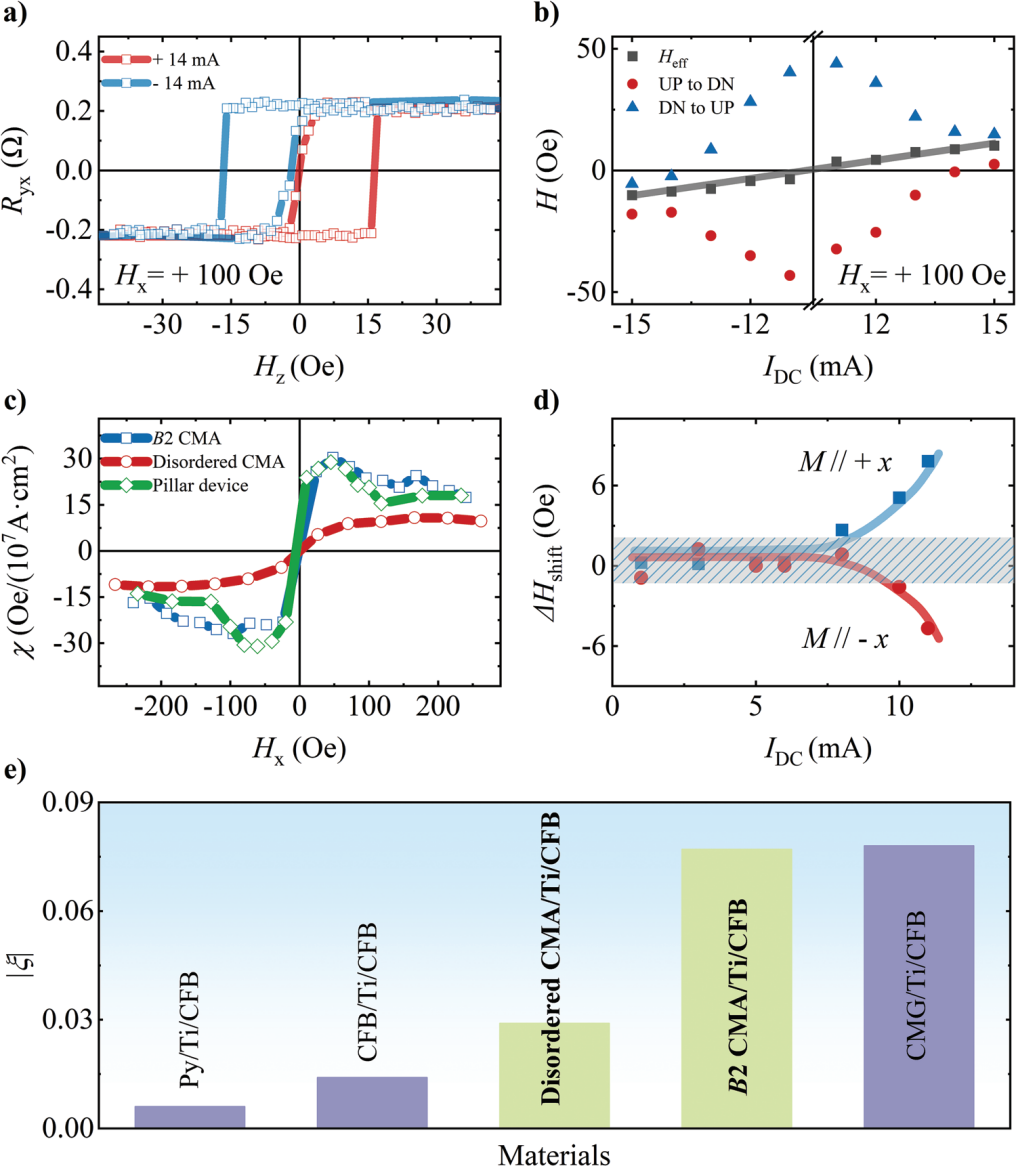
Anomalous Hall resistance loops with *I*_DC_ = ± 14 mA a) and switching fields for down‐to‐up (blue triangles) and up‐to‐down (red dots) magnetization reversals as functions of *I*_DC_ b) in the optimized pillar sample. The black squares in b) represent the center of the anomalous Hall loops. c) Current‐induced effective field per current density as a function of applied in‐plane field for the optimized pillar and normal Hall bar samples. d) Current dependence of the AHE loop shift field measured under zero in‐plane field on the optimized pillar device. The shaded area indicates the minimum detection limits of the gaussmeter. e) Absolute values of spin Hall efficiency in Py/Ti/CFB,^[^ ^38^ ^]^ CFB/Ti/CFB,^[^ ^38^ ^]^ CMG/Ti/CFB.^[^ ^45^ ^]^ and CMAs in the current work.

To quantitatively assess the enhanced SOT efficiency resulting from *B*2 ordering, we performed the current‐induced hysteresis loop shift technique to evaluate the spin Hall efficiency, as $\xi =\frac{4e\mu_{0}M_{s}t_{CFB}}{\pi \hbar }\chi_{SHE}$.^[^ ^44^ ^]^ A clear opposite AHE shift occurred when the direction of *I*_DC_ reversed in *B*2 CMA, as shown in **Figure** **4**a, indicating the presence of a current‐induced effective field, *H*_eff_. Figure 4b shows the trend of *H*_eff_, along with the switching fields for up to down (*H*_UPtoDN_) and down to up (*H*_DNtoUP_) as a function of *I*_DC_. Here,*H*_eff_ =  (*H*_UPtoDN_ + *H*_DNtoUP_)/2, and the switching fields are defined as the zero‐crossing points where *R*_yx_ transitions from positive to negative and vice versa. By extracting the slope of *H*_eff_ under large ± *I*_DC_, we derived the current‐induced effective field per current density *χ* =  *H*_eff_ /*J*_e_ as a function of in‐plane field in Figure 4c. We conducted loop shift measurements on the optimized pillar device, alongside the normal Hall bar, and the *χ* of pillar sample remains nearly the same as that of the Hall bar device. The spin Hall efficiency *ξ* is determined to be 0.077 and 0.029 for the *B*2‐ordered and disordered CMA‐based trilayers, respectively. Moreover, the field‐free spin Hall efficiency in the *B*2 CMA sample has been successfully determined to be 0.029 ± 0.003, as shown in Figure 4d. Nonetheless, we have eliminated the possibility of interlayer coupling as the cause of field‐free switching for several reasons.^[^ ^51^ ^]^ First, Figure 4d shows a threshold current at 8 mA, which contradicts the linear relationship between *∆H*_shift_ and *I*_DC_ typically attributed to stray fields.^[^ ^38^ ^]^ Additionally, a Ti spacer can effectively decouple ferromagnetic layers,^[^ ^52^ ^]^ and studies show that interlayer coupling has minimal influence on current‐induced switching.^[^ ^35^ ^]^ Therefore, the evidence supports that *z*‐spin current is the main driver of field‐free magnetization switching in our system.

Most importantly, our findings underscore the exceptional SOT performance of CMA in comparison to other ferromagnets, as illustrated in Figure 4e. Notably, the spin Hall efficiency of the disordered CMA surpasses that of ferromagnetic CFB (− 0.014) and Py (0.006). Furthermore, the spin Hall efficiency of the *B*2 CMA is amplified twofold compared to the disordered counterpart, even outperforming that of heavy metal Ta (− 0.048).^[^ ^38^ ^]^ In addition, Tang et al. reported a substantial effective spin Hall efficiency *ξ* = − 0.078 in a *B*2‐ordered full Heusler alloy CMG, which is also a ferromagnetic Weyl semimetal.^[^ ^45^ ^]^ This also highlights the excellent SOT performance of Co‐based Heusler alloys.

### 2.4 Strengthening of Spin Hall Efficiency due to Chemical Ordering

Now let us delve into the mechanism of SOT in our Heusler alloy‐based magnetic trilayers. First, it is important to note that the absence of structural gradient^[^ ^39^ ^]^ and inhomogeneous distribution of heavy metal elements in our CMA films, as shown in Figure 1, rules out their contribution to the SOT‐induced magnetization switching. Second, the spin anomalous Hall effect (SAHE), which is the spin counterpart of the AHE in ferromagnets, can also give rise to spin current and has been observed in various materials. For instance, the SAHE has been utilized as a source of SOT in NiFe^[^ ^31^, ^36^ ^]^ and CFB^[^ ^53^ ^]^ films, and has been shown to induce anomalous Hall magnetoresistance in FeMn alloy.^[^ ^43^ ^]^ However, the SAHE can only generate spin current in the transverse direction ***m*** × ***J***_***c***_, where ***m*** is the magnetization of FM. In our measurements, the spin current flows perpendicular to the film plane, while the driving current and auxiliary field are both along ***x***. Thus, the cross product of SAHE contributes zero spin current in the geometry. Third, previous works have suggested that the interfacial spin–orbit filtering is responsible for the generation of spin current in other magnetic trilayer systems.^[^ ^38^ ^]^ However, given that the thickness of the bottom FM layer in this study is much greater than that in previous research, the interfacial effect should be suppressed, so the dominant contribution is from the bulk properties of CMA in our system. Hence, we attribute the large SOT efficiency to the SHE of CMA, which has long been observed in FMs.^[^ ^28^, ^29^, ^30^ ^]^ As for the field‐free switching, previous works have reported that spin current with ***z*** polarization can arise from ***σ*** ∝ ***m*** × ***y*** for charge current flowing in the ***x*** direction due to the interfacial spin–orbit precession in ferromagnet.^[^ ^35^, ^38^, ^41^ ^]^ This effect leads to the reorientation of spin current polarization due to the spin–orbit field near the interface. Very recently, X. R. Wang demonstrated in theory that the spin currents with collinear polarization and propagation can be generated through anomalous spin Hall effect (ASHE) in ferromagnets, following the symmetry of ***J***_s_∝ ***J***_c_ × (***σ*** × ***m***).^[^ ^54^ ^]^ This effect has been recently proven in YIG/Ag/Py trilayers, where a spin current with ***z*** polarization is generated in in‐plane magnetized Py and injected to the adjacent YIG layer, causing the field‐free magnetization switching of perpendicularly magnetized YIG.^[^ ^55^ ^]^ ASHE has now generated interest in the research field, particularly regarding the relationship between energy consumption, the field‐free magnetization switching current threshold, and the out‐of‐plane torque proportion.^[^ ^56^ ^]^ As previously mentioned, the interfacial effects are expected to be weak in our samples due to the significantly thicker CMA films. Consequently, the field‐free switching in our system may be attributed to this anomalous spin Hall effect.

Now we need to understand why the *B*2 CMA has larger spin Hall efficiency than its disordered counterpart. In Heusler alloys, the chemical ordering plays a crucial role in determining various magnetic correlation properties such as the AHE,^[^ ^57^ ^]^ electronic structure,^[^ ^58^ ^]^ magnetic damping constant, and magnetization.^[^ ^59^, ^60^, ^61^ ^]^ It is exactly the reason that we observe larger AHE in *B*2 CMA compared to the disordered counterpart, shown in Figure 2c. Particularly, the CMA has been predicted and experimentally confirmed as a Weyl semimetal,^[^ ^18^, ^20^ ^]^ the band structure can vary significantly depending on the chemical ordering. Therefore, it is reasonable to attribute the enhanced spin Hall efficiency in *B*2 CMA to the formation of chemical ordering and resulted modification of band structure, as compared to the disordered counterpart.

## 3 Conclusion

In conclusion, by fabricating a Heusler alloy CMA‐based magnetic trilayers, the magnetization switching with or without auxiliary magnetic field is achieved in both *B*2 and disordered CMA. Especially, we have observed an enhanced positive spin Hall efficiency *ξ* = 0.077 in *B*2 CMA, surpassing that of the heavy metal Ta − 0.048 in a similar trilayer system. After meticulously eliminating potential effects in ferromagnet, we emphasize the significance of the *B*2‐ordered structure formation in enhancing the spin Hall efficiency. Specifically, this enhancement may stem from the intrinsic presence of the SHE and ASHE in ferromagnet. Ultimately, our discoveries extend the potential applications of Heusler alloys and pave a novel path for enhancing the SOT switching by leveraging the combined effect of SHE and ASHE, thereby advancing the development of devices utilizing ferromagnetic materials.

## 4 Experimental Section

### Sample Preparation and Characterization

The CMA films were deposited on (001)‐oriented MgO single‐crystalline substrates by magnetron sputtering using a Co_2_MnAl target. The *B*2‐ordered CMA were deposited at 450 ^○^C, while the disordered CMA were fabricated at room temperature. In the magnetic trilayers consisting of CMA/Ti/CFB/MgO/SiO_2_, the CFB layer was deposited at room temperature, and serves as a spin‐current analyzer. A Ti spacer layer was deployed to decouple the ferromagnetic layers and develop PMA of CFB, but allows efficient spin current transmission.^[^ ^38^, ^62^ ^]^ The multilayers were then annealed at 150 ^○^C for 1 h in vacuum conditions to promote PMA. The thickness and crystal structure are characterized by X‐ray reflection and XRD techniques with a Bruker D8 Discover diffractometer using Cu Kα radiation (*λ* = 0.15419 nm). The atomic force microscopy measurement is performed with Bruker Dimension Edge atomic force microscope. All Hall bar and pillar structures are patterned using UV lithography with an Ultraviolet Maskless Lithography machine (TuoTuo Technology, UV Litho‐ACA) and Ar ion milling.

### Measurements

The magnetization switching measurements were performed by first applying a 50 µs wide large pulse current using the Keithley 6221 DC and AC current source in the longitudinal direction. Subsequently, the transverse voltage is measured using the Keithley 2182 nanovoltmeter with a 1 ms wide small pulse current. In the anomalous Hall shift measurement, the shift loops were the result of domain expansion or shrinkage, which is a result of the combined contribution of an additional external in‐plane magnetic field and the out‐of‐plane SOT effective field. More details could be found in ref.[45] All measurements were carried out at room temperature.

## Conflict of Interest

The authors declare no conflict of interest.

## Supporting information



Supporting Information

## Data Availability

The data that support the findings of this study are available from the corresponding author upon reasonable request.

## References

[1] N. P. Butch , P. Syers , K. Kirshenbaum , A. P. Hope , J. Paglione , Phys. Rev. B 2011, 84, 220504.

[2] Y. Nakajima , R. Hu , K. Kirshenbaum , A. Hughes , P. Syers , X. Wang , K. Wang , R. Wang , S. R. Saha , D. Pratt , J. W. Lynn , J. Paglione , Sci. Adv. 2015, 1, e1500242. 26601201 10.1126/sciadv.1500242 PMC4640617

[3] J. A. Logan , S. J. Patel , S. D. Harrington , C. M. Polley , B. D. Schultz , T. Balasubramanian , A. Janotti , A. Mikkelsen , C. J. Palmstrom , Nat. Commun. 2016, 7, 11993. 27346655 10.1038/ncomms11993 PMC4931221

[4] K. Sumida , Y. Sakuraba , K. Masuda , T. Kono , M. Kakoki , K. Goto , W. Zhou , K. Miyamoto , Y. Miura , T. Okuda , A. Kimura , Commun. Mater. 2020, 1, 89.

[5] Z. K. Liu , L. X. Yang , S. C. Wu , C. Shekhar , J. Jiang , H. F. Yang , Y. Zhang , S. K. Mo , Z. Hussain , B. Yan , C. Felser , Y. L. Chen , Nat. Commun. 2016, 7, 12924. 27671444 10.1038/ncomms12924 PMC5052656

[6] Z. Wang , M. G. Vergniory , S. Kushwaha , M. Hirschberger , E. V. Chulkov , A. Ernst , N. P. Ong , R. J. Cava , B. A. Bernevig , Phys. Rev. Lett. 2016, 117, 236401. 27982662 10.1103/PhysRevLett.117.236401

[7] R. A. de Groot , F. M. Mueller , P. G. v. Engen , K. H. J. Buschow , Phys. Rev. Lett. 1983, 50, 2024.

[8] K. Özdoğan , E. Şaşıoğlu , B. Aktaş , I. Galanakis , Phys. Rev. B 2006, 74, 172412.

[9] X. Zhu , Y. Dai , C. Luo , J. Magn. Magn. Mater. 2016, 398, 7.

[10] G. H. Fecher , H. C. Kandpal , S. Wurmehl , C. Felser , G. Schönhense , J. Appl. Phys. 2006, 99, 08J106.

[11] R. Yilgin , M. Oogane , S. Yakata , Y. Ando , T. Miyazaki , IEEE Trans. Magn. 2005, 41, 2799.

[12] M. Oogane , T. Kubota , H. Naganuma , Y. Ando , J. Phys. D: Appl. Phys. 2015, 48, 164012.

[13] C. Guillemard , S. Petit‐Watelot , L. Pasquier , D. Pierre , J. Ghanbaja , J. C. Rojas‐Sánchez , A. Bataille , J. Rault , P. Le Fèvre , F. Bertran , S. Andrieu , Phys. Rev. Appl. 2019, 11, 064009.

[14] I. Belopolski , K. Manna , D. S. Sanchez , G. Chang , B. Ernst , J. Yin , S. S. Zhang , T. Cochran , N. Shumiya , H. Zheng , B. Singh , G. Bian , D. Multer , M. Litskevich , X. Zhou , S. M. Huang , B. Wang , T. R. Chang , S. Y. Xu , A. Bansil , C. Felser , H. Lin , M. Z. Hasan , Science 2019, 365, 1278. 31604235 10.1126/science.aav2327

[15] J. Noky , Y. Zhang , J. Gooth , C. Felser , Y. Sun , npj Comput. Mater. 2020, 6, 77.

[16] J.‐C. Tung , G.‐Y. Guo , New J. Phys. 2013, 15, 033014.

[17] K. Manna , L. Muechler , T.‐H. Kao , R. Stinshoff , Y. Zhang , J. Gooth , N. Kumar , G. Kreiner , K. Koepernik , R. Car , J. Kübler , G. H. Fecher , C. Shekhar , Y. Sun , C. Felser , Phys. Rev. X 2018, 8, 041045.

[18] J. Kübler , C. Felser , Europhys. Lett. 2016, 114, 47005.

[19] A. Hoffmann , IEEE Trans. Magn. 2013, 49, 5172.

[20] P. Li , J. Koo , W. Ning , J. Li , L. Miao , L. Min , Y. Zhu , Y. Wang , N. Alem , C. X. Liu , Z. Mao , B. Yan , Nat. Commun. 2020, 11, 3476. 32651362 10.1038/s41467-020-17174-9 PMC7351740

[21] Y. Sakuraba , K. Hyodo , A. Sakuma , S. Mitani , Phys. Rev. B 2020, 101, 134407.

[22] Y. M. Ji , W. X. Zhang , H. B. Zhang , W. L. Zhang , New J. Phys. 2022, 24, 053027.

[23] A. Manchon , J. Zelezny , I. M. Miron , T. Jungwirth , J. Sinova , A. Thiaville , K. Garello , P. Gambardella , Rev. Mod. Phys. 2019, 91, 035004.

[24] N. Zhao , A. Sud , H. Sukegawa , S. Komori , K. Rogdakis , K. Yamanoi , J. Patchett , J. W. A. Robinson , C. Ciccarelli , H. Kurebayashi , Phys. Rev. Mater. 2021, 5, 014413.

[25] C. Ciccarelli , L. Anderson , V. Tshitoyan , A. J. Ferguson , F. Gerhard , C. Gould , L. W. Molenkamp , J. Gayles , J. Železný , L. Šmejkal , Z. Yuan , J. Sinova , F. Freimuth , T. Jungwirth , Nat. Phys. 2016, 12, 855.

[26] L. Zhu , X. S. Zhang , D. A. Muller , D. C. Ralph , R. A. Buhrman , Adv. Funct. Mater. 2020, 30, 2005201.

[27] T. Seki , K. Uchida , T. Kikkawa , Z. Qiu , E. Saitoh , K. Takanashi , Appl. Phys. Lett. 2015, 107, 092401.

[28] J. Cramer , A. Ross , S. Jaiswal , L. Baldrati , R. Lebrun , M. Kläui , Phys. Rev. B 2019, 99, 104414.

[29] W. L. Yang , J. W. Wei , C. H. Wan , Y. W. Xing , Z. R. Yan , X. Wang , C. Fang , C. Y. Guo , G. Q. Yu , X. F. Han , Phys. Rev. B 2020, 101, 064412.

[30] D. Tian , Y. Li , D. Qu , S. Y. Huang , X. Jin , C. L. Chien , Phys. Rev. B 2016, 94, 020403(R).

[31] W. Wang , T. Wang , V. P. Amin , Y. Wang , A. Radhakrishnan , A. Davidson , S. R. Allen , T. J. Silva , H. Ohldag , D. Balzar , B. L. Zink , P. M. Haney , J. Q. Xiao , D. G. Cahill , V. O. Lorenz , X. Fan , Nat. Nanotechnol. 2019, 14, 819. 31332346 10.1038/s41565-019-0504-0 PMC11566720

[32] Y. W. Oh , J. Ryu , J. Kang , B. G. Park , Adv. Electron. Mater. 2019, 5, 1900598.

[33] Y. Hibino , T. Taniguchi , K. Yakushiji , A. Fukushima , H. Kubota , S. Yuasa , Nat. Commun. 2021, 12, 6254. 34716327 10.1038/s41467-021-26445-y PMC8556288

[34] T. Seki , Y.‐C. Lau , S. Iihama , K. Takanashi , Phys. Rev. B 2021, 104, 094430.

[35] J. Ryu , R. Thompson , J. Y. Park , S.‐J. Kim , G. Choi , J. Kang , H. B. Jeong , M. Kohda , J. M. Yuk , J. Nitta , K.‐J. Lee , B.‐G. Park , Nat. Electron. 2022, 5, 217.

[36] T. Y. Ma , C. H. Wan , X. Wang , W. L. Yang , C. Y. Guo , C. Fang , M. K. Zhao , J. Dong , Y. Zhang , X. F. Han , Phys. Rev. B 2020, 101, 134417.

[37] T. Seki , S. Iihama , T. Taniguchi , K. Takanashi , Phys. Rev. B 2019, 100, 144427.

[38] S. C. Baek , V. P. Amin , Y. W. Oh , G. Go , S. J. Lee , G. H. Lee , K. J. Kim , M. D. Stiles , B. G. Park , K. J. Lee , Nat. Mater. 2018, 17, 509. 29555998 10.1038/s41563-018-0041-5

[39] M. Tang , K. Shen , S. Xu , H. Yang , S. Hu , W. Lu , C. Li , M. Li , Z. Yuan , S. J. Pennycook , K. Xia , A. Manchon , S. Zhou , X. Qiu , Adv. Mater. 2020, 32, 2002607. 10.1002/adma.202002607 32596934

[40] Z. Luo , Q. Zhang , Y. Xu , Y. Yang , X. Zhang , Y. Wu , Phys. Rev. Appl. 2019, 11, 064021.

[41] V. P. Amin , J. Zemen , M. D. Stiles , Phys. Rev. Lett. 2018, 121, 136805. 30312060 10.1103/PhysRevLett.121.136805

[42] K. S. Das , W. Y. Schoemaker , B. J. van Wees , I. J. Vera‐Marun , Phys. Rev. B 2017, 96, 220408(R).

[43] Y. Yang , Z. Luo , H. Wu , Y. Xu , R. W. Li , S. J. Pennycook , S. Zhang , Y. Wu , Nat. Commun. 2018, 9, 2255. 29884868 10.1038/s41467-018-04712-9 PMC5993777

[44] C. F. Pai , M. Mann , A. J. Tan , G. S. D. Beach , Phys. Rev. B 2016, 93, 144409.

[45] K. Tang , Z. Wen , Y.‐C. Lau , H. Sukegawa , T. Seki , S. Mitani , Appl. Phys. Lett. 2021, 118, 062402.

[46] L. Leiva , S. Granville , Y. Zhang , S. Dushenko , E. Shigematsu , T. Shinjo , R. Ohshima , Y. Ando , M. Shiraishi , Phys. Rev. B 2021, 103, L041114.

[47] L. Neumann , M. Meinert , AIP Adv. 2018, 8, 095320.

[48] L. Zhu , Adv. Mater. 2023, 35, 2300853. 10.1002/adma.202300853 37004142

[49] L. Liu , C. Zhou , X. Shu , C. Li , T. Zhao , W. Lin , J. Deng , Q. Xie , S. Chen , J. Zhou , R. Guo , H. Wang , J. Yu , S. Shi , P. Yang , S. Pennycook , A. Manchon , J. Chen , Nat. Nanotechnol. 2021, 16, 277. 33462431 10.1038/s41565-020-00826-8

[50] Q. Yang , D. Han , S. Zhao , J. Kang , F. Wang , S.‐C. Lee , J. Lei , K.‐J. Lee , B.‐G. Park , H. Yang , Nat. Commun. 2024, 15, 1814. 38418454 10.1038/s41467-024-46113-1 PMC10901790

[51] Y.‐C. Lau , D. Betto , K. Rode , J. M. D. Coey , P. Stamenov , Nat. Nanotechnol. 2016, 11, 758. 27240416 10.1038/nnano.2016.84

[52] S. S. Parkin , Phys. Rev. Lett. 1991, 67, 3598. 10044776 10.1103/PhysRevLett.67.3598

[53] S. Iihama , T. Taniguchi , K. Yakushiji , A. Fukushima , Y. Shiota , S. Tsunegi , R. Hiramatsu , S. Yuasa , Y. Suzuki , H. Kubota , Nat. Electron. 2018, 1, 120.

[54] X. R. Wang , Commun. Phys. 2021, 4, 55.

[55] M. Yang , L. Sun , Y. Zeng , J. Cheng , K. He , X. Yang , Z. Wang , L. Yu , H. Niu , T. Ji , G. Chen , B. Miao , X. Wang , H. Ding , Nat. Commun. 2024, 15, 3201. 38615046 10.1038/s41467-024-47577-x PMC11016059

[56] T. Zhang , C. Wan , X. Han , Phys. Rev. B 2023, 108, 014432.

[57] E. Vilanova Vidal , H. Schneider , G. Jakob , Phys. Rev. B 2011, 83, 174410.

[58] T. Graf , C. Felser , S. S. P. Parkin , Prog. Solid State Chem. 2011, 39, 1.

[59] P. J. Webster , Contemp. Phys. 1969, 10, 559.

[60] P. J. Webster , R. S. Tebble , Philos. Mag. 1967, 16, 347.

[61] R. Mandal , I. Kurniawan , I. Suzuki , Z. Wen , Y. Miura , T. Kubota , K. Takanashi , T. Ohkubo , K. Hono , Y. K. Takahashi , ACS Appl. Nano Mater. 2022, 5, 569.

[62] Y. W. Oh , S. H. Chris Baek , Y. M. Kim , H. Y. Lee , K. D. Lee , C. G. Yang , E. S. Park , K. S. Lee , K. W. Kim , G. Go , J. R. Jeong , B. C. Min , H. W. Lee , K. J. Lee , B. G. Park , Nat. Nanotechnol. 2016, 11, 878. 27428279 10.1038/nnano.2016.109 PMC11279531

