# Correction to “Large Spin Hall Efficiency and Current‐Induced Magnetization Switching in Ferromagnetic Heusler Alloy Co_2_MnAl‐Based Magnetic Trilayers”

**DOI:** 10.1002/advs.202504185

**Published:** 2025-03-20

Wang M, Pan C, Xie N, et al. Large Spin Hall Efficiency and Current‐Induced Magnetization Switching in Ferromagnetic Heusler Alloy Co_2_MnAl‐Based Magnetic Trilayers. *Advanced Science*, 2025, 12(4): 2407171.

The *y*‐axis unit in Figure 3c is incorrectly labeled as “Oe.” It should be “Ω” (ohms), as this figure represents the anomalous Hall resistance of the device, a quantity measured in ohms.


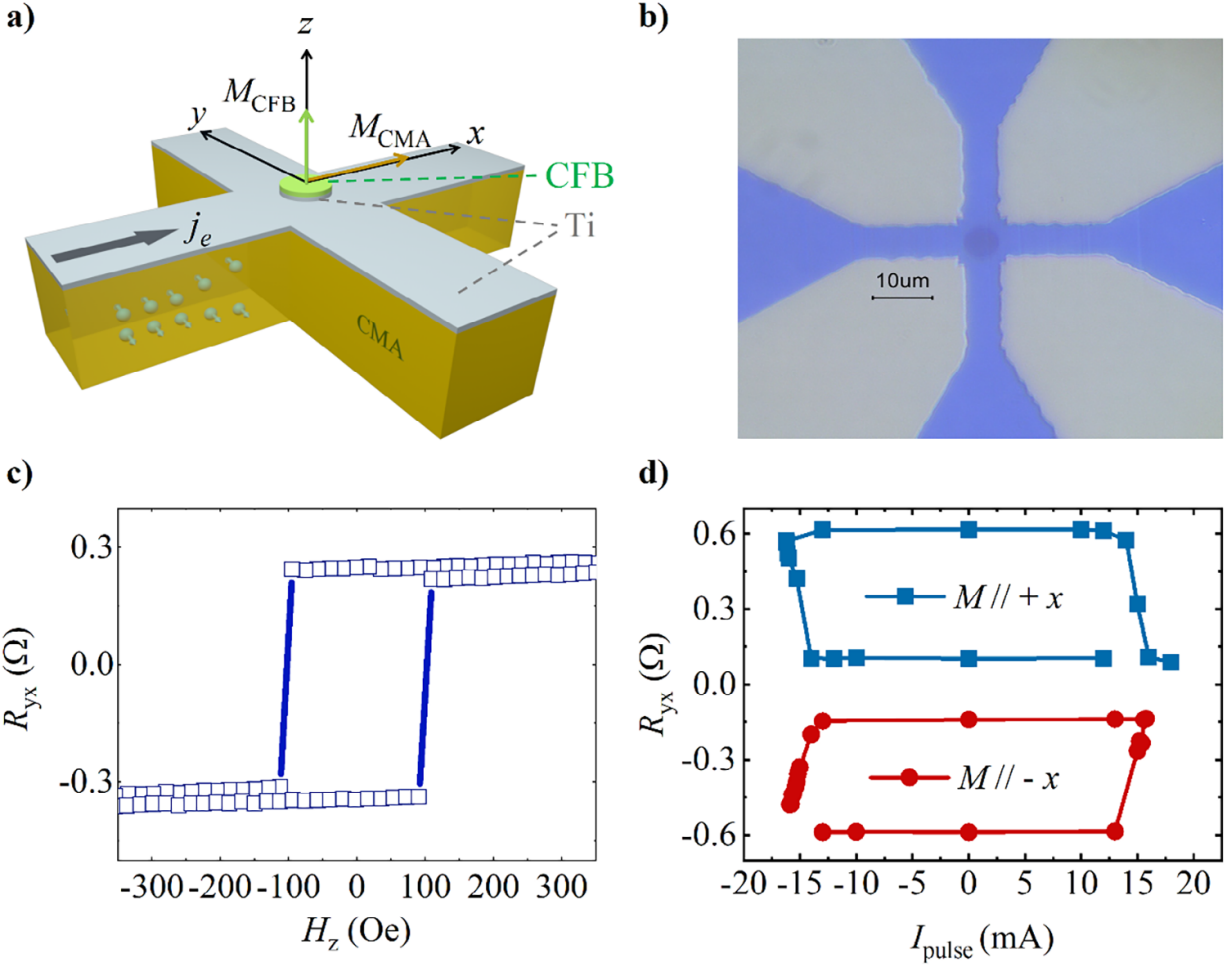


We sincerely apologize for this error.

