# Supplementary material for: Large Spin Hall Efficiency and Current‐Induced Magnetization Switching in Ferromagnetic Heusler Alloy Co2MnAl‐Based Magnetic Trilayers
Source: Adv Sci (Weinh). 2024 Dec 4;12(4):2407171. doi: 10.1002/advs.202407171 (PMC12121694; doi:10.1002/advs.202407171)
Supplement: Supplementary file 1 — Supporting Information [file ADVS-12-2407171-s001.docx]

Supporting Information

**Large Spin Hall Efficiency and Current-induced Magnetization Switching in Ferromagnetic Heusler Alloy Co_2_MnAl-based Trilayers**

Mingzhi Wang, Chang Pan, Nian Xie, Xuepeng Qiu*, Yufei Li, Lili Lang, Shiqiang Wang, Dashuai Cheng, Weijia Fan*, Shi-Ming Zhou, Zhong Shi*

**S1. Current-induced magnetization switching in disordered CMA-based trilayer**

Current-induced magnetization swithcing with or without external auxiliary magnetic field are demonstrated in disordered CMA-based trilayer, as well. Consistent with the *B*2 CMA case, the polarity of switching is reversed when a positive and negative auxiliary field is applied (Fig. S1.1) or when the magnetization of disoredered CMA is align with $+ x$ and $- x$ direction (Fig. S1.2). The effective spin Hall efficiency is also positive.

Fig. S1.1. Current-induced magnetization switching in disordered CMA-based trilayer under different auxiliary magnetic field ranging from $+ 180 \mathrm{Oe}$ to $- 180 \mathrm{Oe}$.

Fig. S1.2. a) and b) are the field-free switching after aligning the magnetization of disordered CMA to the positive and negative $\mathbf{x}$ direction.

**S2. M-H loop and negligible current induced magnetization switching of Ti/CFB/MgO layers**

Fig. S2.1 displays the out-of-plane magnetic hysteresis loop of the Ti (1.5)/CFB (0.9)/MgO (1.5) multilayers. The magnetization is evaluated to be $1520 \mathrm{emu}/\mathrm{cc}$.

For clarification, we conducted current-induced magnetization switching under auxiliary magnetic field. In Fig. S2.2, the strong PMA of Ti/CFB/MgO is evidenced by the anomalous Hall resistance. However, we did not observe consistent and reproducible current-induced magnetization switching, suggesting the lack of SOT without the presence of CMA. This finding is consistent with a previous report.^[38]^

Fig. S2.1. Out-of-plane magnetic hysteresis loop of the Ti (1.5)/CFB (0.9)/MgO (1.5) multilayers

Fig. S2.2. a) anomalous Hall resistance and b) Current-induced magnetization switching of the Ti (1.5)/CFB (0.9)/MgO (1.5) multilayers. The auxiliary magnetic field is set to ± 180 Oe.
